# Regulation of tRNA expression during the social cycle of the amoeba *Dictyostelium discoideum*

**DOI:** 10.1186/s12860-026-00580-0

**Published:** 2026-03-07

**Authors:** Dulce I. Valdivia, Peter F. Stadler

**Affiliations:** 1 Department of Computer Science & Interdisciplinary Center for Bioinformatics, Bioinformatics Group, Leipzig University, Härtelstraße 16–18, D-04107 Leipzig, Germany; 2 Max Planck Institute for Mathematics in the Sciences, Inselstraße 22, D-04103 Leipzig, Germany

**Keywords:** Transfer RNAs (tRNA), Non-coding RNA, Translation, Genome regulation, Social cycle, *Dictyostelium discoideum*

## Abstract

Transfer RNAs (tRNAs) are the decoders of the protein coding genetic information, as they transfer amino acids into nascent proteins during messenger RNA translation. This pivotal role makes tRNAs a source of translation regulation that can affect protein synthesis. Still, we are beginning to understand the upstream mechanisms regulating tRNA pools themselves. In *Dictyostelium discoideum*, starvation of a sufficient number of individuals, triggers the development of a coordinated sporulation response denominated the social cycle. By using publicly available transcriptomic, epigenomic, and genomic data, we studied two factors contributing to the regulation of tRNA pools throughout this social cycle. First, the tRNA gene repertoire shows that the compact genome of *D. discoideum* escapes translational selection as even with a relatively high number of tRNA genes, anticodon and codon frequencies greatly mismatch. This disparity is explained by the overrepresentation of anticodons that can be modified in the wobble position. During the social cycle, the vast majority of tRNA genes lie in nucleosome free regions, indicating that most genes are always contributing to the tRNA pools. However, there is a marked variation in expression levels of the proteins involved in tRNA maturation. This modulation is ultimately mirrored by fine-tuned differential composition of tRNA pools at isodecoder, isoacceptor and isotype levels. Particularly, there is an overall down-regulation in the vegetative to streaming transition. Key elements bypass this down-regulation pattern and taken together, this evidence suggests compensatory mechanisms in tRNA regulation that might rescue translation for the following developmental stages, thus allowing *D. discoideum* to evolve this remarkable strategy under the pressure of an amino acid scarce environment.

**Supplementary Information:**

The online version contains supplementary material available at 10.1186/s12860-026-00580-0.

## Background

Transfer RNAs (tRNAs) are the mediators of the genetic code, linking the genetic information expressed in a messenger RNA (mRNA) to its protein product by means of antiparallel anticodon:codon pairings [1]. Protein production rates, therefore, are not solely determined by mRNA abundances but also depend critically on tRNA concentrations and the composition of the tRNA pool (reviewed e.g. in [2]).

In a neutral scenario, the tRNA supply is steady and sufficient for all cellular conditions. This would imply that the composition of the tRNA pool is determined exclusively by the frequency and organization of the tRNA genes and, thus, protein abundance would be determined by mRNA abundance and regulatory mechanisms acting on translation that are unrelated to tRNA availability [3, 4]. The emergence of tRNA sequencing coupled protocols (e.g. ARM-seq [5], QuantM-tRNA seq [6], LOTTE-seq [7], mim-tRNAseq [8]; reviewed in [9]) to assess tRNA transcript diversity and abundance have shown that this is not the case. There is ample evidence that tRNA pools in fact are condition-dependent and variations can alter protein synthesis and generate aberrant phenotypes [6, 10–13]. Thus, tRNAs are, at the very least, a relevant part of the regulatory network that modulates protein synthesis and may even contribute to epistatic or pleiotropic phenomena.

The composition of the tRNA pool depends on two main factors: the tRNA gene repertoire, which explains all possible transcripts, and the tRNA biosynthesis pathway, explaining the temporal changes in tRNA availability.

Transfer RNA genes are subject to frequent duplication events that tend to create tandem arrays distributed throughout the genome and multiple gene copies that bear identical – or almost identical – sequences [14, 15]. Although, in principle, a full set of tRNA anticodons would only need 61 genes, eukaryotic cells on average have about 400 tRNA genes [16]. Some species exhibit dramatic deviations from this number. For example, in vertebrates, birds have an average of 169 tRNA genes while amphibians have 1229 tRNA genes on average [17]. The zebrafish *Danio rerio* harbors about 20,000 tRNA genes within its 1.6 Gb genome [10, 14]. Despite this overrepresentation of tRNAs copies across genomes, eukaryotic species are generally lacking tRNA genes encoding for 16 anticodons [2, 18]. This set includes a subset of eight anticodons that are frequently absent in the whole Tree of Life. Moreover, some phenotypes seem to depend on specific tRNA genes, as other active gene copies encoding the same tRNA may lead to protein mistranslation and, therefore, are not functionally equivalent [19].

To explain the expansion of tRNA genes, some factors have been found to be widely correlated with tRNA copy number. These include genome size [14, 16], number of protein coding genes [16], amino acid usage [20, 21] and codon usage [22–24]. The initial correlations with codon usage led to the formulation of the translational selection hypothesis, which posits that codon usage evolves by adaptation to the tRNA pools. Later on, it was proposed that tRNA copy number and genome size interact to define a landscape where translational selection can operate [24]. However, as it is often the case [25], early studies and even modern large-scale ones have been conducted on datasets with a high percentage of bacterial species, and only a small and phylogenetically limited set of eukaryotes.

The production of mature tRNAs is a complex process that is subject to multiple layers of regulation. Although tRNAs are only 70–90 bp long, their biosynthesis requires epigenetic and post-transcriptional processes. To start with, tRNA genes are shorter than a complete DNA wrap around a histone ($\sim$140 bp) and, therefore, to be they must lie on nucleosome free regions (NFR) [26, 27]. tRNAs are transcribed by the RNA polymerase III (PolIII) which seems to read at least some of the active histone modifications of protein-coding genes (e.g. H3K4me3 and H3K27ac) [28]. All tRNA genes include two internal promoters, the A-box and B-box, which are recognized by the assembly factor TFIIIC (gene *gtf3c5*). After binding, TFIIIC recruits the transcription factor TFIIIB (gene *tbpA*) and positions it upstream the transcription start site of the tRNA gene. In turn, TFIIIB recruits the RNA polymerase III to start transcription. Other aspects, such as the genome topology and proximity to RNA polymerase II [28–30], may contribute to the initiation of transcription.

Once a primary tRNA transcript is synthesized, distinct RNases cleave out the 5’ leading and 3’ trailer sequences [31]. In some cases, tRNAs contain introns that will be removed by excising the intronic sequence and ligating the resulting exons [32]. Mature tRNAs have a 3’-end CCA tail that is attached by a tRNA nucleotidyltransferase, the CCA-adding enzyme. At this mature tail, the corresponding aminoacyl-tRNA synthetases will catalyze the transfer of the amino acid [33]. As an additional layer, tRNAs are decorated with an extensive variety of chemical modifications that are essential to stabilize the tertiary structure, to be recognized by the transport proteins and to properly couple with ribosomes during protein synthesis [34]. Modifications in the first nucleoside of the anticodon create wobble pairings that enable tRNAs to decode non-complementary codons [35]. Ultimately, wobble pairing mechanisms are the counterpart of the degeneracy on the third codon position in the genetic code [36]. The differential expression of individual tRNA genes is reflected by the abundance of tRNA-derived fragments (tRFs). The comparative analysis of tRF and mature tRNA levels thus may help to disentangle transcriptional and post-transcription regulation in tRNA maturation [37].

Multicellularity is a convergent trait that has arisen at least 16 times during eukaryotic evolution [38]. Two types of multicellularity are distinguished: clonal, found in metazoans and embryophytes, where cells originate from divisions of a single founder cell; and aggregative, where cells with likely different genotypes unite [38]. The amoeba *Dictyostelium discoideum* exhibits the most refined example of aggregative multicellularity in eukaryotes [39]. This life cycle is called the “social cycle”, the “multicellular sporocarpic cycle” [39], or “developmental morphogenesis” [40]. It is a dormancy strategy driven by starving a sufficient number of amoebas, primarily due to amino acid restriction [41, 42]. The social cycle is characterized by distinct stages [39, 40, 43]: First, up to $10^5$ starved amoebas secrete cyclic adenosine monophosphate (cAMP) as a chemoattractant to create a multicellular *aggregate*. Once they have aggregated, the amoebas start *streaming* toward a central domain and form a dense *mound*. Later, the mound elongates into a finger-shaped sorogen. Interestingly, the sorogen can fall over and migrate extensively to find a favorable location in which to establish the fruiting body. This mobile stage is called a *slug*. Slugs show similar features to embryogenesis in clonal multicellularity: they differentiate into four cell types (pre-spore, pre-stalk, cup, and basal disc cells) and display a well-defined anterior–posterior pattern. After approximately 24 hours of starvation, slugs culminate in sorocarps, or *fruiting bodies*, which are composed of a stalk and a sorus. The stalk consists of vacuolated stalk and basal disc cells, while the sorus consists of spores and cup cells. The compact spores are encased in a triple-layered cell wall. Only amoebas that differentiate into spores will germinate and grow into new individuals when conditions are favorable; the rest will die.

Since 80% of the amoebae become dormant spores [40], one could hypothesize that tRNA expression decreases as cells acquire their final identities within the fruiting body. Therefore, in this study, we aim to explore how the two upstream factors, tRNA repertoire and tRNA biogenesis, shape tRNA pools during the social cycle of *D. discoideum*. To do so, we make use of publicly available sequencing datasets [44, 45] that allow us to describe the encoded tRNA repertoire, to follow the changes in transcriptional accessibility tRNA genes (ATAC-seq), the expression levels of genes involved in tRNA biogenesis (RNA-seq) and the expression levels of mature tRNAs (LOTTE-seq).

We found that instead of having a diverse anticodon repertoire that reflects codon usage in protein coding genes, *D. discoideums*’s genome has expanded a limited set of anticodons that can be chemically modified for wobble decoding. Most tRNA genes are transcriptionally accessible throughout the cycle, meaning that tRNA diversity is generally stable. Unexpectedly, genes involved in tRNA biogenesis and, in agreement, tRNAs, are down-regulated in the beginning of the cycle rather than at the end. Furthermore, our results suggest that tRNA availability during the social cycle is maintained by a few elements that might compensate for the suppressed biosynthesis pathway.

## Methods

### Data collection

The reference genome A×4 v2.7 of *Dictyostelium discoideum*, coding sequences (CDS) and gene annotation were obtained from the NCBI database.

We retrieved publicly available sequencing data from two distinct publications. In [44], chromatin accessibility and gene expression were assessed by ATAC-seq and RNA-seq experiments at the following stages of the social cycle: vegetative (0 hours - starvation), streaming (6 hours), mound (10–12 hours), slug (15–18 hours), and culmination to fruiting body (22 hours). In [45], mature tRNA levels were assessed by LOTTE-seq experiments at the following stages: vegetative (0 hours - starvation), streaming (6 hours), slug (16 hours), and culmination to fruiting body (20 and 24 hours). For our analysis, we used the ATAC-seq and RNA-seq libraries from four stages: vegetative (0 hours), streaming (6 hours), mound (10–12 hours), and culmination to fruiting body (22 hours) (see Fig. 4a). From the LOTTE-seq data, we used the libraries with the greatest overlap: vegetative (0 h), streaming (6 h), and culmination to fruiting body (20 h and 24 h) (see Fig. 5a). For simplicity, we refer to samples from the beginning of culmination to fruiting body establishment as “fruiting body”. Libraries corresponding to 15–18 hours (slugs) from both publications were excluded because sorogens in axenic strains are more likely to proceed directly to culmination than those in non-axenic strains [40], and the oldest samples would in fact overlap with the expected onset of culmination. This could reduce the resolution between the compared developmental time points. All the accessions and their corresponding developmental stages can be consulted in Supplementary Methods Table S1.

### ATAC-seq data processing

**Fig. 1:**
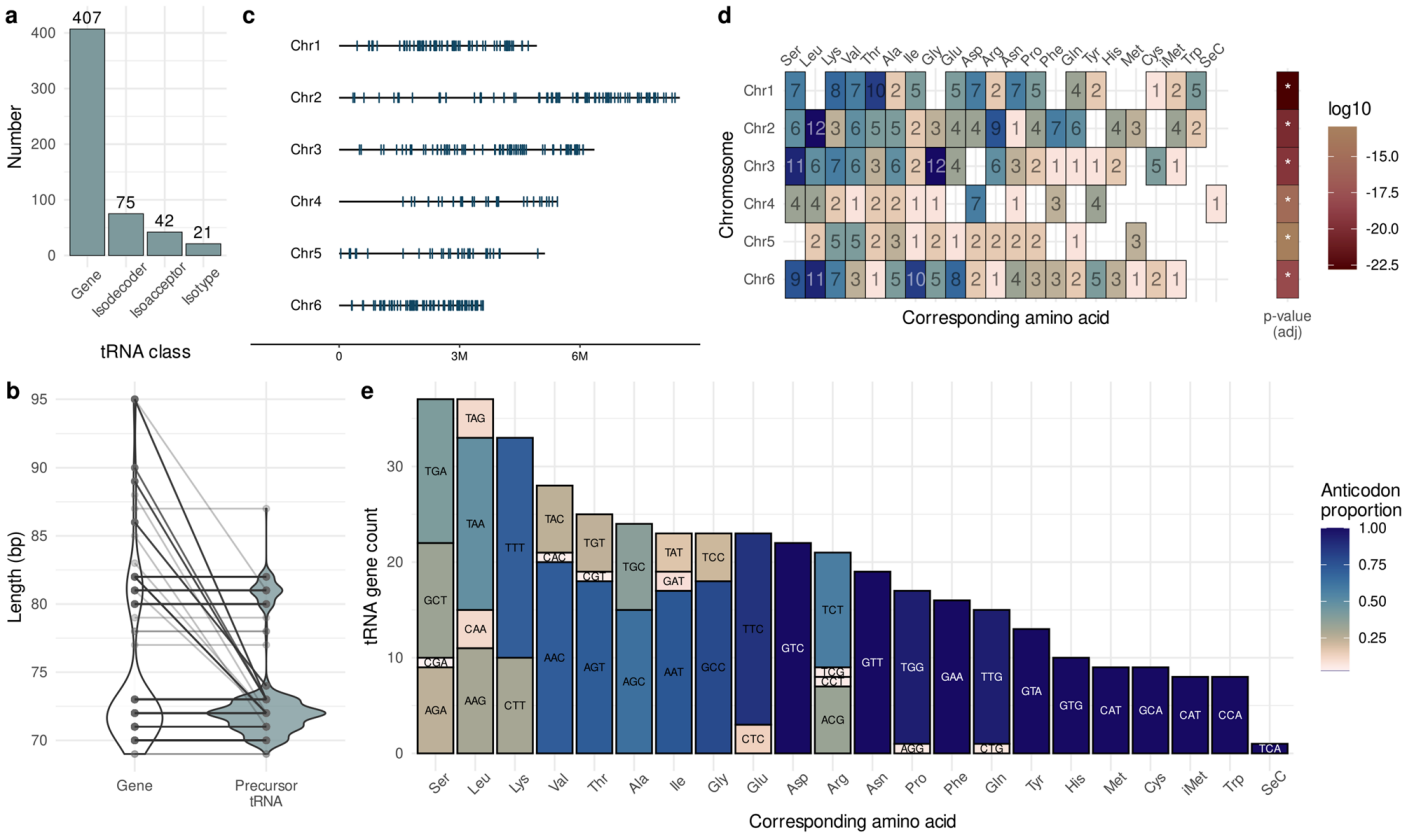
Genomic landscape of the tRNA gene repertoire in *Dictyostelium discoideum*. (**a**) Number of tRNA genes and tRNAs categorized as isodecoders, isoacceptors, or isotypes. (**b**) Length distribution of tRNA genes and their corresponding precursors (after intron removal). Each line connects a tRNA gene with its processed tRNA. (**c**) The genomic localization of tRNA genes across the six chromosomes. (**d**) Left: number of tRNA isotypes in each chromosome. Right: *p*-values from the multivariate hypergeometric test for each chromosome, showing deviations from random isotype localization. (**e**) tRNA gene counts by isotype, divided by anticodon. The color fill within each anticodon bar represents its proportion of the total gene count for that isotype

We followed the ENCODE processing pipeline recommendations. **Quality check and trimming.** The quality of the raw libraries (150 bp, paired-end) was evaluated using fastQC v0.11.4 [46] before and after trimming. Adapters and low quality bases were trimmed using cutadapt v1.16 [47]. **Mapping.** Trimmed reads were mapped to the *D. discoideum* genome assembly A×4 v2.7 using bowtie2 v2.5.3 [48, 49], with the following parameters: -X 2000 –very-sensitive –dovetail –no-discordant –fr. **Filtering.** Properly mapped paireds were selected with samtools v1.21 [50]. Duplicated reads were discarded using picard’s utility MarkDuplicates v2.5.0 [51], as well as reads mapping to the mitochondrial genome. **Fragment distribution assessment**. We corroborated the presence of nucleosome-free regions (NFR) and different nucleosome positioning patterns across the experiments (Sup. Methods Fig. 1a) **Peak calling.** Reads were shifted and length selected using deepTool’s [52] utility alignmentSielve v3.5.4.post1 with parameters: –ATACshift –maxFragmentLength 100. Peak calling was performed on each replica using Macs2 v2.1.1.20160309 [53], with parameters: –nomodel –shift 75 –extsize 100 –keep-dup all –call-summits –bdg. Peak reproducibility between all replicates, was assessed by computing the pairwise Irreproducibility Discovery Rate (IDR) [54] with the R package IDR v2.0.4.2 (Sup. Methods Fig. 1b). **Consensus peaks**. To generate a high-confidence peak set for each stage, we first performed IDR analysis on the merged replicates of each stage to create an “oracle peak set”. Then, pairwise reproducible peaks (IDR $\leq$ 0.05) were identified in a second round of IDR analysis using the corresponding oracle peak set as an additional input. Lastly, these pairwise reproducible peaks per stage were merged. High-confidence peak sets were validated for read counts correlation and reproducibility using the R package DiffBind [55, 56]. Only the peaks from the best two replicates of the mound stage were used in the downstream analyses (Sup. Methods Fig. 1c-e). **tRNA loci in nucleosome free regions.** We considered a tRNAs gene as transcriptionally available if according to BEDTools’s v2.31.1 [57] utility intersect, its coordinates were fully embedded in a high-confidence NFR peak.

### RNA-seq data processing

**Quality check and trimming.** Quality check of the raw libraries was evaluated using fastQC v0.11.4 [46] before and after trimming. Adapter sequences and low quality bases were trimmed with Trimmomatic v0.36 [58]. **Mapping.** Trimmed reads were mapped against *D. discoideum* genome using STAR 2.7.11b [59]. **Differential expression analysis.** Read count was performed with featureCounts v1.4.6-p5 [60]. Raw counts were processed with the R package edgeR [61] for library normalization and differential expression analysis using a generalized model. Differentially expressed genes were selected using the decideTest function which applies the Bonferroni-Holms *p*-value normalization.

### Identification of proteins involved in tRNA biosynthesis

Genes involved in tRNA biosynthesis were identified using QuickGO Gene Ontology and GO Annotations from EMBL-EBI (https://www.ebi.ac.uk/QuickGO/annotations). We restricted the search to *D. discoideum* and download the following terms: GO:0006400 (tRNA modification), GO:0042797 (tRNA transcription by RNA polymerase III), GO:0002097 (tRNA wobble base modification), GO:0006383 (transcription by RNA polymerase III), GO:0001680 (tRNA 3’-terminal CCA addition), GO:0099116 (tRNA 5’-end processing) and GO:0043039 (tRNA aminoacylation). As the search for genes annotated with tRNA 3’-end processing did not produce any results in this database, we manually expanded this search on dictyBase (http://dictybase.org/). This last search retrieved one 3’-end processing gene (*rnZ*) and extra subunits of the 5’-end processing complex.

### LOTTE-seq data processing

We based our pipeline on the best practice workflow for tRNA sequencing data developed by [15]. It was implemented in the following manner: **Reference tRNA sequences**. A *de novo* tRNA gene discovery was performed using tRNAscan-SE v2.0 [62]. The predicted gene sequences were extracted from the genome using BEDTools’s v2.31.1 [57] utility getfasta, with the intron removal option enabled. To obtain the set of unique tRNA sequences (i.e., isodecoders), identical sequences were clustered. A reference sequence for each cluster was created by appending two distinct sequences: at the 5’-end, we added the 10 bp upstream sequence of an arbitrary tRNA gene within the same cluster, and a “CCA” at the 3’-end to simulate mature tRNAs. **Masked genome**. Predicted tRNAs genes were hard masked with BEDTools’s utility maskfasta. **Quality check and trimming**. Library quality of the LOTTE-seq experiments (100 bp, single-end) was assessed before and after trimming using fastQC v0.11.4 [46]. Adapters were trimmed and reads shorter than 8 bp were discarded with cutadapt v1.16 [47]. **Mapping**. Clean reads were divided into two groups based on their length: short reads (8–21 bp) and long reads ($\geq$ 22 bp). Long reads were mapped in two steps. First, they were mapped against the masked genome using segemehl v0.3.4 [63–65] with stringent parameters: –evalue 500 –differences 3 –maxinterval 1000 –accuracy 80. Unmapped reads were saved, while reads that mapped to the masked genome were discarded. The unmapped reads were then mapped to the reference tRNA cluster sequences using the same segemehl settings. For downstream analysis, only reads satisfying all the following criteria were retained: (i) uniquely mapped (tag ‘NH:i:1’), (ii) the leftmost mapping position is located after the end of the artificial flanking region, (iii) had a CCA 3’-end and, (iv) the mapping extends to the 3’-end of the mature tRNA reference sequence. These filters ensured that the reads were valid LOTTE-seq reads, as they reflect the experimental CCA-tail selection, reverse transcription, and computational adjustments. Short reads match with reverse transcription premature stops (caused by chemical modifications), and due to their small size, they are prone to map randomly across the genome. To minimize the loss of these reads, short reads were directly mapped against the tRNA clusters (same segemehl parameters) and we retain only those that satisfy the (i)-(iv) criteria described above. **Differential expression analysis.** Reads were count directly from the bam files after filtering. We used edgeR [61] for library normalization and to test for differential expression at isodecoder level (equivalent to cluster counts). For isoacceptors and isotypes differential expression, we added the counts of all the isodecoders in the corresponding group. In all cases, differentially expressed genes were selected using the decideTest function which applies the Bonferroni-Holms *p*-value normalization.

### Data analysis and graph construction

All statistical analyses as well as the implementation of the decoding potential graph were done in R v4.3.2 [66] and Rstudio v2023.12.1.402 [67], using different packages: igraph [68, 69], ggraph [70], tidyverse [71], scico [72].

## Results

### Codon usage is predominantly explained by non-canonical pairings in the absence of many anticodons

Transfer RNAs are grouped into equivalence classes according to their behaviour at three distinct functional levels. In a genome-to-protein view: tRNAs that are encoded in distinct genes but lead to identical mature sequences are called “isodecoders”. In transcriptomic analyses, isodecoders are also termed “clusters”, as they represent identical transcripts that are counted together as they cannot be distinguished. Then, tRNAs that have the same anticodon are grouped as “isoacceptors”, though they might exhibit variation in the rest of their sequences. Finally, “isotypes” are tRNAs that will be charged with the same amino acid.

The nuclear genome of *Dictyostelium discoideum* contains 407 tRNA genes that mature into only 75 distinct isodecoders, which in turn encode 42 distinct anticodons (i.e., 42 isoacceptors; Fig. 1a). These tRNAs are still potentially capable of charging all 20 standard amino acids plus selenocysteine (i.e., 21 tRNA isotypes). The length of nuclear tRNAs exhibits a bimodal distribution, ranging from 69 to 95 bp as genes and from 69 to 87 nt after intron removal (Fig. 1b). Although tRNAs are spread throughout the entire genome (Fig. 1c), the distribution of tRNA gene copies across chromosomes is not random. For instance, the smallest chromosome, chr6, has the highest tRNA gene density (Sup. Table S1). Furthermore, when examining gene copy numbers for each amino acid, we observe that certain isotypes are particularly expanded in some chromosomes (Fig. 1d, left). A multivariate hypergeometric test shows that the distribution of isotype copy numbers in each chromosome deviates significantly from random localization (Fig. 1d, right). It is worth noting that eight of the nuclear tRNA genes correspond to the special initiator tRNA (iMet^CAT^). Additionally, there are 18 mitochondrial tRNA genes encoding 16 amino acids; the remaining mt-tRNAs are most likely imported from nuclear tRNAs [73, 74].

**Fig. 2:**
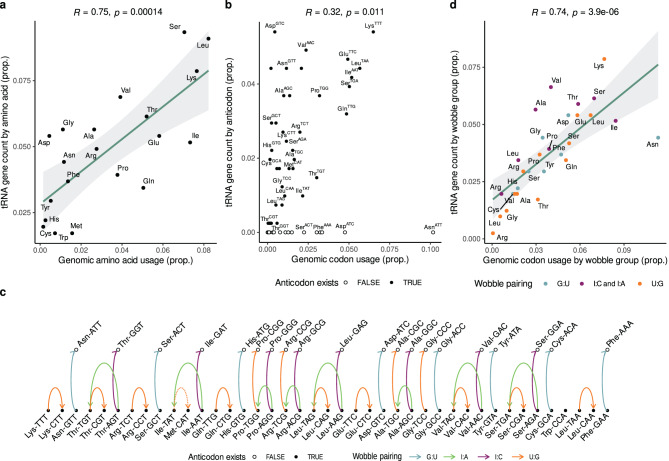
Codon usage is well explained by potential wobble pairings of the tRNA repertoire (**a**) Strong and positive Pearson correlation between amino acid frequency in the genomic coding sequences and tRNA gene copy numbers grouped by isotype. (**b**) The strength of this Pearson correlation is lost when coding sequences are analyzed by codon frequency and the tRNA gene copy numbers are grouped by anticodons (isoacceptors). Only points with sufficient spacing are labeled with their amino acid and anticodon. See larger version in sup. Fig. 1c. (**c**) The decoding potential graph for *D. discoideum*. The vertex set includes all the possible anticodons. Missing anticodons are shown on the top layer (non-filled dots), while existing anticodons are shown in bottom layer (filled black dots). A directed edge (arrow) indicates that the anticodon at the tail of the arrow can translate the same codon that the anticodon at the head of the arrow via wobble interactions. The wobble pairings required for this replacement are marked with a color: blue (G:U), orange (U:G), green (I:A) or pink (I:C). Dotted arrow denotes the potential non-synonymous amino acid replacement. (**d**) Pearson correlation of tRNA gene copy numbers and codon frequency of anticodons grouped by the edges of the decoding potential graph. Thus, the color of each point represents the wobble pairing that connects them in the graph. I:C and I:A counts are considered together because they come from the same anticodons

The protein-coding sequences of *D. discoideum* utilize the complete set of 62 codons (considering the special use of the stop codon UGA for selenocysteine), but only 42 anticodons were found, there are 20 anticodons that are not encoded by any tRNA gene. To assess whether the nuclear tRNA gene repertoire can, in principle, satisfy the genomic codon usage, we analyzed the correlation between codon usage and tRNA gene copy numbers at both isotype and isoacceptor levels. As expected, the number of isotypes showed a strong and positive Pearson correlation ($R=0.77$, *p*-value $=1.4\cdot 10^{-4}$) with genomic amino acid usage (Figure 2a). However, this correlation decreases dramatically ($R=0.32$, *p*-value $=0.011$) when examined at the finer level of isoacceptors (Fig. 2b; see enlarged version in Sup. Fig. 1). Here, we observe that some anticodons such as Asp^GTC^ are overrepresented in the genome even if their corresponding codons are rarely used. Conversely, some abundant codons in *D. discoideum*’s genome, including the most abundant codon AAT (which pairs with Asn^ATT^), lack an encoded cognate tRNA.

**Fig. 3:**
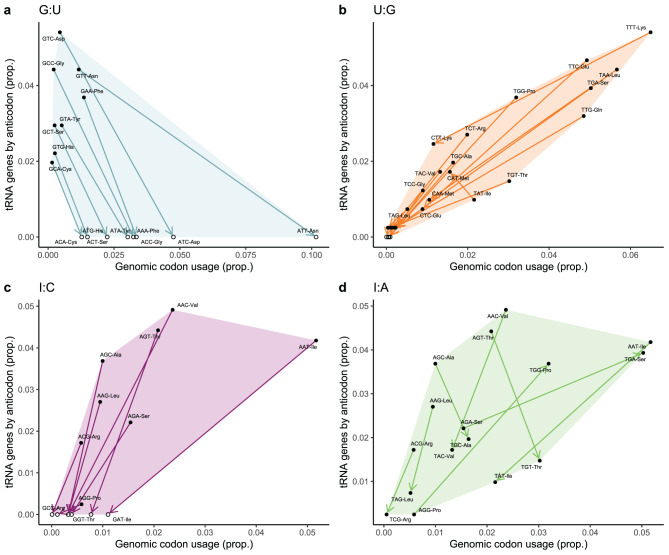
Decoding potential graph divided by wobble pairing type. Decoding potential graph overlaid on tRNA gene copy number versus genomic codon usage for each wobble pairing type. Each wobble pairing type shows a characteristic arrow direction (e.g., top-left to bottom-right) and the spatial area spanned by these interactions. Black points represent anticodons that are encoded in the genome, while open white points are missing anticodons. (**a**). G:U pairings (*top-left to bottom-right*): existing anticodons with higher genomic representation than their cognate codons in coding sequences compensate for missing anticodons with frequent cognate codons. (**b**). U:G pairings (*top-right to bottom-left; exception: Ile*^*TAT*^ →*Met*^*CAT*^): anticodons with high copy number and higher codon usage compensate for those with few copies and infrequent cognate codons. Spanning mostly the center diagonal. (**c**) I:C pairings (*direction: top-right to bottom-left*), anticodons with high copy number and frequent cognate codons compensate for missing anticodons with infrequent cognate codons. (**d**) I:A pairings (*top-right to bottom-left; exceptions: Ser*^*AGA*^ → * Ser*^*TGA,*^* Ala*^*AGC*^ → * Ala*^*TGC*^*, Pro*^*AGG*^ → * Pro*^*TGG*^): existing anticodons compensate other existing anticodons with smaller copy number. Spanning a broader central area than U:G

**Fig. 4:**
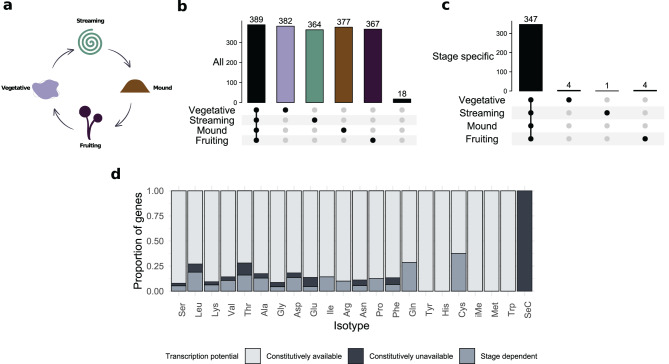
tRNA transcription availability throughout the social cycle. (**a**). Diagram depicting the stages of the social cycle (vegetative, streaming, mound and fruiting) that were analyzed with ATAC-seq data. (**b**). Upset plot (union). Each bar shows the number of tRNA genes that lie within NFR in at least one of the stages marked with a black circles bellow each bar. Gray circles indicate that the stage is not considered. Only 18 tRNA genes did not overlapped with a NFR at any of the analyzed stages. (**c**). Upset plot (intersection). Each bar shows the number of tRNA genes that appear within an NFR at all stages marked with a black circle. Only a few tRNA genes are specific to a single stage: four are vegetative-specific, one is streaming-specific, and four are mound-specific. No fruiting-specific tRNAs were detected. (**d**). Proportion of genes in the distinct transcriptional categories grouped by isotype

**Fig. 5:**
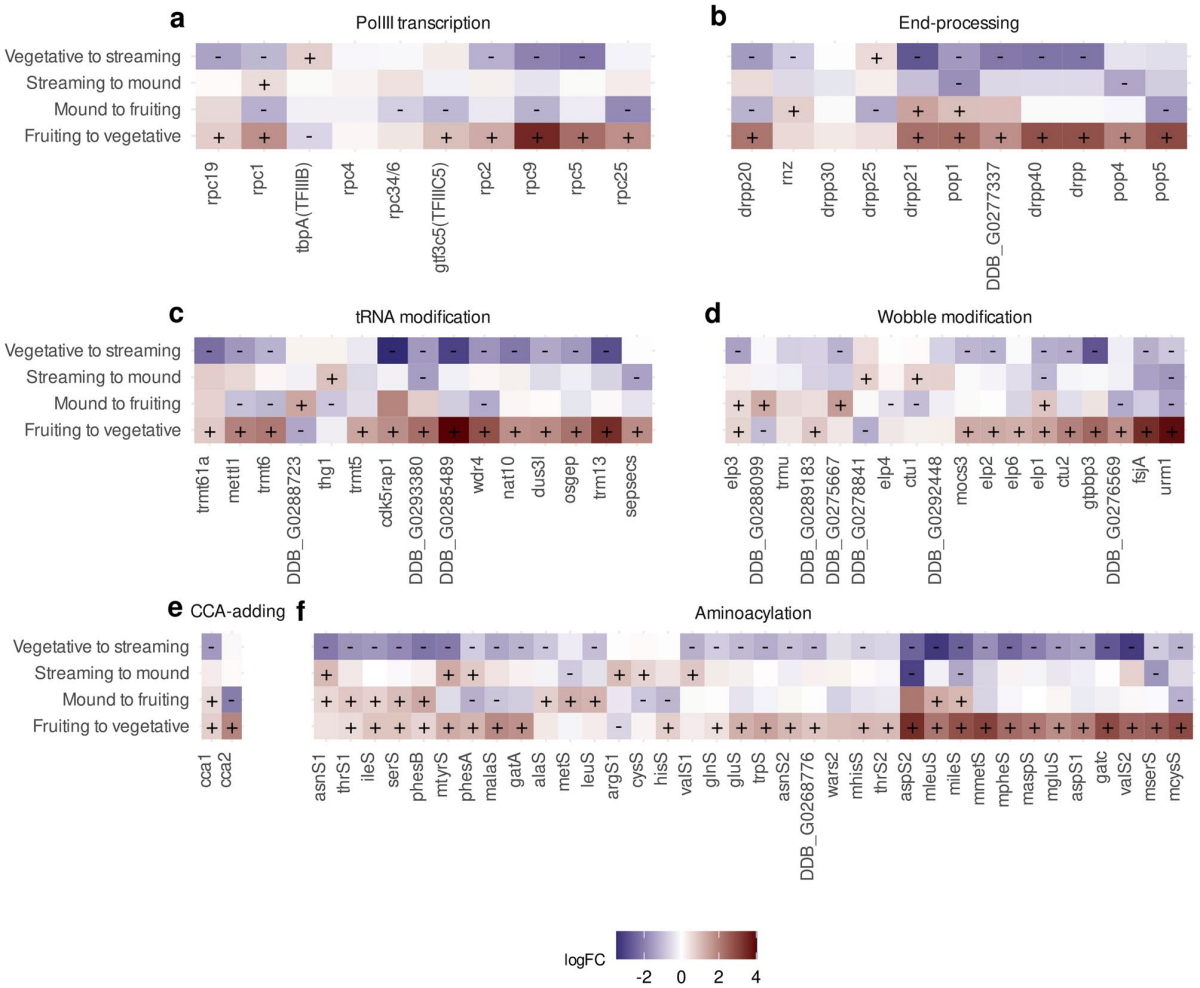
Expression patterns of tRNA biosynthesis pathway genes. Differential expression of genes involved in (**a**) Transcription by RNA polymerase III, (**b**) 5’-end and 3’-end tRNA processing, (**c**) tRNA modification enzymes, (**d**) 3’-end CCA addition, (**e**) tRNA wobble base modification, and (**f**) tRNA aminoacylation. The symbols “+” and “-” indicate that a gene is statistically significantly up- or down-regulated, respectively. The fill color represents the log-fold change (logFC) of gene expression between two stages

Following Crick’s wobble hypothesis [35, 75, 76], an inosine at position 34 may pair with A, U and C; moreover G:U and U:G pairs may be utilized in codon:anticodon interactions. In tRNAs, these types of non-canonical recognition are further supported by extensive chemical modifications [36], see also [77] for empirical evidence in *Dictyostelium*. To account for non-complementary decoding in a systematic manner, we introduce the *decoding potential graph* to describe which anticodon has the potential to decode the cognate codon of another one, provided that the proper wobble pairing is possible. More precisely, the vertex set of this graph comprises the 61 possible anticodons and the edges indicate which anticodon can replace another one during translation. Furthermore, we divide the vertices into two sets: the anticodons that exist in the genome and those that are missing. We note that selenocysteine was excluded from this analysis to avoid introducing spurious stop-codon counts coming from gene annotation. Consider the following example (see diagram in Sup. Fig. 2): the anticodon ATT (reverse complement on the tRNA UUA) decodes the codon AAT (AAU in the mRNA). If the anticodon ATT does not exist in the tRNA repertoire, then the anticodon GTT (UUG) could also decode AAT, assuming a G:U wobble pairing. Thus, in the decoding potential graph, we insert an arc from GTT to ATT, colored for the G:U wobble pairing. More abstractly, consider two anticodons $a_1$ and $a_2$, where $a_1$ is the standard anticodon for the codon $c_1$. If there is a wobble pairing $w$ such that $w(a_2)$ pairs with $c_1$, then we set an arc $a_2 \rightarrow a_1$ with color $w$. In this graph, we observe that these four types of wobble decoding create different connection patterns (Fig. 2c). For instance, G:U and I:C only connects existing anticodons with missing anticodons. These two types of pairings alone can compensate 16 of the missing anticodons. The remaining four missing anticodons can be decoded by U:G pairings, which additionally connects existing anticodons with missing anticodons. Lastly, I:A pairings only connect existing anticodon pairs. It is worth noting that the only non-synonymous amino acid replacement that can occur is Ile^TAT^ decoding the codon of Met^CAT^ by means of a G:U wobble pairing. Then, we used the *decoding potential graph* to group tRNA gene copy numbers and codon usages of anticodons that are directly connected in the graph. We observe that this strategy reestablishes the correlation observed at the isotype level ($R=0.74$; *p*-value$=3.9\cdot 10^{-6}$)(Fig. 2c).

Lastly, we aimed to investigate how the *decoding potential graph* can reestablish the anticodon-codon correlation. To do so, we overlaid the *decoding potential graph* on the isoacceptor copy number versus codon-usage plot (i.e., Fig. 2b). We observe that each type of wobble pairing resolves specific sets of abnormal anticodon expansion and codon usage patterns and are restricted to specific areas of the plot (Fig. 3a-d). In general, the direction of all the arrows goes from top to bottom, indicating that anticodons capable of wobble decoding are preferentially expanded in *D. discoideum*. Furthermore, individual anticodon groupings by each wobble pairing type exhibit strong correlations (Sup. Fig. 3). For instance, anticodons that compensate for others via G:U show a correlation coefficient of $R=0.94$ (*p*-value $=1.1\cdot10^{-6}$).

In summary, we observe a strong correlation between codon usage and tRNA gene frequency in *D. discoideum* only if we account for wobble-based, non-complementary modes of decoding.

### Most tRNA genes are available for transcription throughout the social cycle

To determine how much of *D. discoideum*’s tRNA gene repertoire has the potential to be transcribed and, therefore, contribute to the tRNA pool during the social cycle, we investigate which tRNA genes are located in nucleosome-free regions (NFRs) by using ATAC-seq experiments performed at four stages of the cycle (vegetative, streaming, mound and fruiting; Fig. 4a). Because mitochondrial reads are excluded in ATAC-seq experiments, we continue to focus our analysis on nuclear encoded tRNAs.

We found that almost all tRNA genes (95.5%, 389 out of 407) are transcriptionally available in at least one stage of the cycle (Fig. 3b). Most of these genes (89.2%, 347 out of 389) are consistently located in NFR across all the evaluated stages, making them constitutively available for transcription. This group of genes include all tRNA isotypes for methionine, tyrosine, histidine and tryptophan (Fig. 3c). Although stage-specific genes are rare, tRNAs genes for cysteine and glutamic acid isotypes have the highest proportion of stage-dependent regulation.

tRNA genes that are not located within NFRs in any of the analyzed stages are likely to be constitutively repressed during the social cycle. Surprisingly, the only gene encoding selenocysteine tRNAs appears to be constitutively repressed across all stages analyzed here. This may be a consequence of limited demand since *D. discoideum* expresses only five selenoproteins [78, 79], each of which contains a single selenocysteine. The three sequences annotated as pseudogenes by tRNAscan-SE [62] also fall in this category. For one of these pseudogenes, its anticodon could not be identified. The other two, however, correspond to the most abundant isotype families, serine and leucine. Besides, their anticodons, Ser^TGA^ and Leu^TAA^, are among the most expanded within their respective isoacceptor families (Fig. 1e). Considering their gene family background, these findings suggest two hypotheses: first, these pseudogenes may reflect isoacceptors that have reached their copy number limit, beyond which further expansion is no longer advantageous. Alternatively, relaxed selection allows the expansion and sequence variation of these isoacceptors; driven by the need to satisfy the previously demonstrated asymmetric anticodon-codon demand as well as the distinct modification patterns [45]. In both cases, a layer of epigenetic regulation can be expected. In the first case, histone-mediated repression blocks the transcription machinery to prevent the expression of spurious tRNAs. In the latter case, these genes may represent new isodecoders that are still in the process of being integrated into the genome’s regulatory framework.

### Silencing of tRNA biosynthesis pathway genes and decrease of mature tRNA levels marks entry to the social cycle

In light of our previous results, we asked whether tRNA transcriptional, post-transcriptional processing and the final mature tRNA pool remain stable during the same four stages of the social cycle examined in the previous section. Therefore, to check if there is any change between the four stages, we focused on three major transitions: (i) from the vegetative stage to the streaming stage, (ii) from the streaming stage to the mound stage, (iii) from the mound stage to the fruiting stage, and (iv) the reactivation of the amoebas when they transition from the fruiting body stage back to the vegetative stage.

We first analyzed the changes in the expression patterns of proteins involved in tRNA biosynthesis by focusing on five functional groups of genes: (1) transcription by RNA polymerase III (PolIII), (2) 5’-end and 3’-end tRNA end processing, (3) tRNA modifications, (4) tRNA terminal CCA addition and (5) tRNA aminoacylation. Remarkably, we observed a consistent down-regulation of most of these genes during the first transition from vegetative to streaming state (Fig. 5a-e). Interestingly, the group of genes that are not silenced during this transition includes those encoding POlIII transcription factors *tbpA* (TFIIIB) and *gtf3c5* (TFIIIC5), the recently described secondary gene copy of the CCA-adding enzyme *cca2* [80], genes involved in uridine wobble modifications (*elp1*, *elp6* and *ctu1*) and four genes coding aminoacyl-tRNA synthetases. The subsequent stages exhibit a more diverse regulatory pattern: while downregulation of some genes becomes even more pronounced, other genes, in contrast, regain at least part of their expression and become significantly upregulated.

**Fig. 6:**
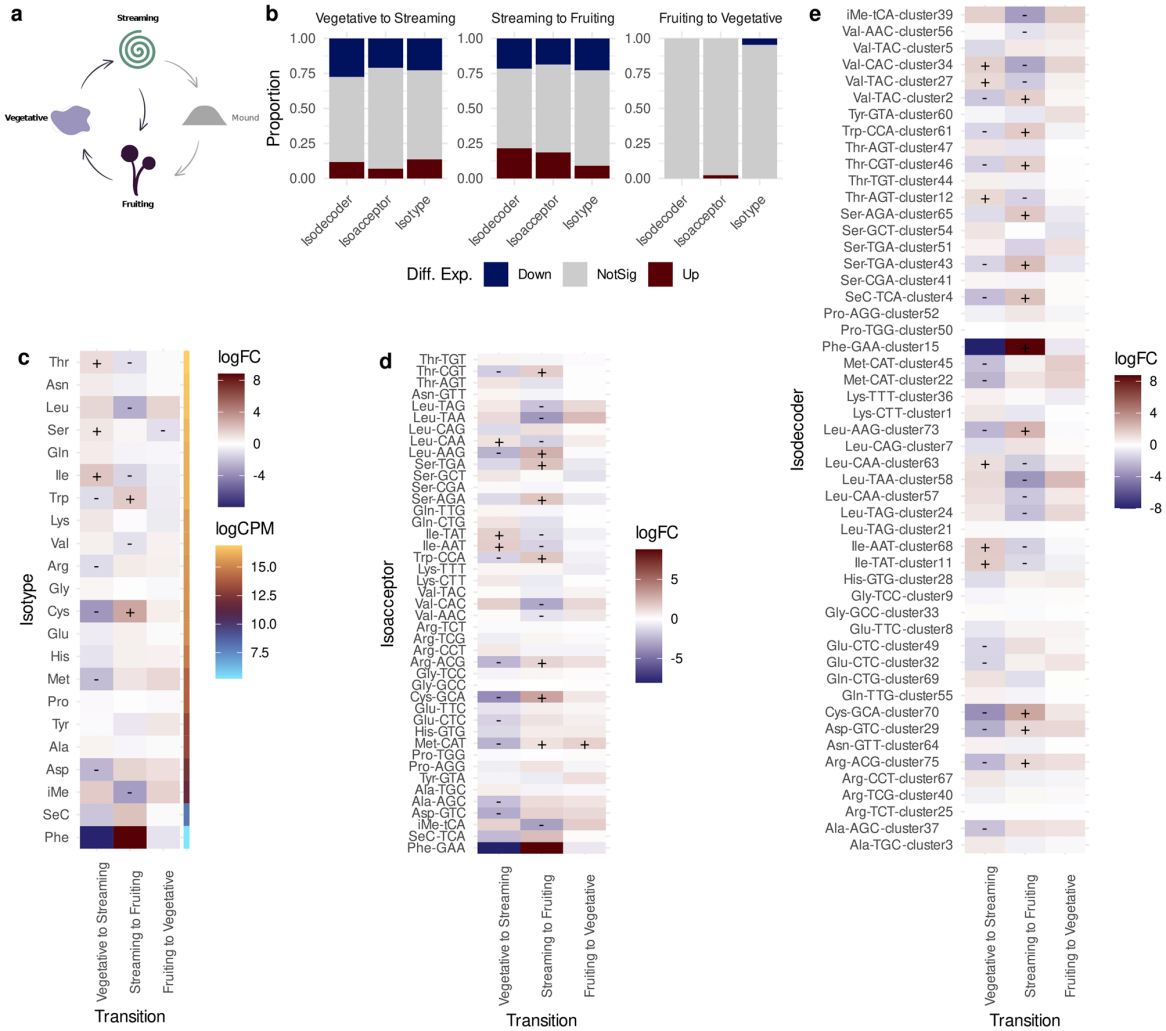
Expression levels of mature tRNAs during the social cycle of D. discoideum (**a**) Diagram depicting the three stages analyzed using LOTTE-seq. In comparison with the previous sections, the mound stage is not evaluated here. (**b**) Proportion of mature tRNAs exhibiting differential expression levels, shown according to their tRNA class and cycle transition (raw counts are shown in sup. Table S2). Expression patterns of mature tRNA isotypes (**c**), isoacceptors **d**. And isodecoders (**e**). The symbols “+” and “-” indicate that a tRNA is statistically significantly up- or down-regulated, respectively. The fold change of tRNA expression logarithmic between two stages (logFC) is indicated by the fill color. As a reference of the abundance of each tRNA isotype in the tRNA pool, we show in c the read count expressed in logarithmic counts per million scale (logCPM) during the vegetative state

Lastly, we analyzed LOTTE-seq data to investigate the variation in expression of mature tRNAs (i.e., tRNA transcripts with the 3’-end CCA tail) across three transitions of the social cycle where the sequencing datasets overlapped (Fig. 6a; see Methods): (i) vegetative to streaming stages, (ii) streaming to fruiting stages, and (iii) fruiting to vegetative stages. Before testing for differential expression, we noticed that all but three nuclear isodecoder sequences (51 out of 54) had a sufficient number of read counts to be included in the differential expression analysis and we further confirmed that sequences corresponding to pseudogenes were not detected in mature tRNA sequences. The proportion of differentially expressed mature tRNAs shows that the tRNA pool has more variation at isodecoder level than isoacceptors and the overall isotype composition (Fig. 6b). Thus, regulation is focused at this finer level where the anticodon:codon interactions are needed during translation and, particularly, where sequence specificity might be required for chemical modifications. For example, during the first transition, variation in valine tRNAs as isotype (Fig. 6c) or Val^TAC^ as isoacceptor (Fig. 6d) is not detectable because the two isodecoders Val^TAC^-cluster5 and Val^TAC^-cluster2 have opposite regulation patterns than Val^TAC^-cluster27 (Fig. 6e).

Consistently with the down-regulation of proteins involved in tRNA biosynthesis during the first transition from vegetative to streaming stages, we observe the highest proportion of mature tRNA isodecoders with decreased abundance in this same transition (Fig. 5b). Comparing within the corresponding tRNA classes, this transition also exhibits the lowest proportion of mature tRNAs with significant increase of expression (Fig. 5b). Interestingly, although the special tRNA iMet^tCA^, which is needed for the initiation of translation, is not differentially expressed, it shows an over-expression trend in this transition and it becomes significantly repressed in the next transition of the social cycle (Fig. 5e).

Later on, the transition from streaming to the development of the fruiting body, exhibits the greatest variation in the tRNA pool composition across all tRNA classes. Particularly, this transition shows the highest proportion of increased mature tRNA levels, exceeding even the changes observed during the restoration of the vegetative state. This finding is in good agreement with the changes in expression levels observed in the tRNA biogenesis pathway, where many proteins become up-regulated and other are further repressed.

## Discussion

In this work we dissected two main factors contributing to tRNA pools during the social cycle of *D. discoideum*: the tRNA gene repertoire and the changes in the regulation of the tRNA biogenesis pathway.

First, we found that the tRNA gene repertoire in *D. discoideum* has a restricted anticodon set, whose frequencies are only weakly correlated to codon usage. This was unexpected, given that as isotypes, tRNA genes exhibited a strong correlation with amino acid usage and, in principle, the combination of tRNA copy number (407 genes) and genome size (34 Mb) places *D. discoideum* in the region of maximal translational selection according to the “theoretical landscape” proposed in [24]. Wobble pairings expand the decoding potential of a tRNA and, in genomes under translational selection, it is expected that these non-canonical pairings make only a minor contribution to mRNA decoding, as codon usage in protein-coding regions should reflect the available anticodon gene repertoire. In the case of *D. discoideum*, constructing its decoding potential graph enabled us to see that wobble pairings, along with—probably selective—tRNA gene duplications, explain the genomic codon usage, particularly in the absence of many anticodons (Fig. 2). For instance, using the decoding potential graph, we saw that—with only four exceptions—it is always the case that overrepresented anticodons (i.e., those with more copies than expected from their codon usage) are the ones that can be chemically modified to create wobble pairings (Sup. Fig. 3) and are likely to replace the missing or underrepresented cognate anticodons in amino acid delivery during translation.

The analysis of nucleosome-free regions showed that 87.7% of the tRNA genes 89.2% of tRNA genes were constantly available for transcription. This arrangement contrasts with results from mammals, whose cells exhibit smaller fractions of available tRNA genes. For example, a computational model, which takes into account the genomic context of tRNA genes, predicted that only three quarters of the tRNA genes are active in at least one cell type [27]. Consistent with this computational result, slightly more than a third of human tRNA genes appeared to be constantly expressed in differentiated hiPSC cells, while another third was sometimes repressed [11]. In addition, the latter study found that although ATAC-seq was a weak predictor of mature tRNA levels, the signal of tRNA genes in the NFR strongly overlapped with the ChIP-seq signal of the Pol III catalytic core component RCP1 [11]. This confirms that ATAC-seq signal can be used as a qualitative indicator of tRNA transcription rather than a quantitative one (i.e., whether a tRNA gene is transcribed or not, rather than how much it is expressed) and, therefore, this method suggests that most tRNA genes in *D. discoideum* are, in fact, transcribed. This constant arrangement of tRNAs in NFRs in *D. discoideum*, moreover, fits well with the absence of A/B compartments (euchromatin/heterochromatin regions) observed during the first transition in the social cycle [81].

Although most tRNA genes are accessible for transcription, we observed a down-regulation of most proteins involved in tRNA biosynthesis during the first transition of the social cycle (Fig. 5). Consistently, there was also a reduction of mature tRNAs at isodecoder and isotype levels (Fig. 5). Taking together, these two results suggest that tRNA pools are mostly modulated by post-transcriptional processes rather than by the transcriptional activation or repression of tRNA genes.

These results contradict our initial hypothesis that there would be a decrease in mature tRNAs at culmination when the surviving amoebas form spores. Phylogenetic studies support the hypothesis that the common ancestor of Dictyostelia only formed solitary cysts, as this trait is conserved across three of its four inner clades [39]. Therefore, instead of delaying tRNA silencing until spore formation in the fruiting body, we could alternatively propose that the overall silencing is part of the ancestral encystation response. Since it has been reported that over half of the proteins with a statistically significant change in abundance increase in the first transition [82], our results suggest the existence of compensatory mechanisms that maintain the tRNA pool and prevent full dormancy, allowing the social cycle to begin. These include: (1) the transcription factors TFIIIC (no change in expression) and TFIIIB (up-regulated), consistent with the fact that the recruitment of PolIII can be maintained after the first transcription cycle only with TFIIIB [83]; (2) previously reported secondary CCA-adding enzyme (CCA2), which is inversely regulated compared to its paralog, CCA1, and its expression level peaks during the first hours of the social cycle [80]; (3) most tRNA genes continue to contribute to the tRNA pool and the special isodecoder iMet at least maintains its expression level.

The use of independent datasets, in which collection times and general laboratory conditions may differ, represents a limitation of this study as it introduces additional variance into the measurements. At the same time, it provides confidence that the results and patterns identified as significant are robust and not driven by dataset-specific effects. Further experiments performed under tightly controlled conditions will likely yield more refined and stronger signals. For instance, such experiments will be necessary to determine whether tRNA abundance is strongly reactivated in the transition of spores to the vegetative state, as it would be expected from the parallel dynamics of the differential expression analysis of the tRNA biogenesis machinery (e.g. see Sup. Fig. 4 for a complementary analysis), and to better understand the link between known pre-starvation sensing elements, such as PSF [39], and tRNA regulation. This link is relevant as early studies showed that amino acid starvation is the main factor triggering the social cycle in *D. discoideum*, in contrast to glucose, which had only a modest effect delaying aggregation [41]. In yeast, GCN2 (General control nonderepressible 2 protein) senses amino acid depletion through its HisRS domain, which is highly similar to histidyl-tRNA synthetases and thus recognizes uncharged tRNAs. GCN2 then inhibits translation by phosphorylating eIF2$\alpha$ (elongation initiation factor 2$\alpha$) [84]. In *D. discoideum*, there are two eIF2$\alpha$ kinases, IfKA and IfKB, which share most of the domain architecture of GCN2, including the HisRS domain. Notably, it was shown that IfKA normally phosphorylates eIF2$\alpha$ from 1 h to 7 h after the onset of the social cycle [42], suggesting that uncharged tRNAs are more abundant at this time than immediately after removal of the food source.

Finally, on a broader scale, a comprehensive picture of the evolutionary forces shaping tRNA usage will require systematic studies of the tRNA pools and tRNA biogenesis pathways in a phylogenetically broad range of unicellular eukaryotes. Extreme cases, such as dinoflagellates that usually featuring exceptionally large genomes ($\geq$ 200 GB) or which are not packaged by histones [85] or *Entamoeba histolytica*, which possesses around $4,500$ tRNA genes and has been reported to harbor a high load of laterally transferred genes [86, 87] may well have adapted their tRNA system in very different ways.

## Conclusion

A detailed reanalysis of publicly available data reveals a complex interplay of codon usage and tRNA copy numbers of tRNA genes, highlighting the importance of wobble pairings in the genome evolution of the social amoeba *D. discoideum*. Furthermore, by tracing tRNA biogenesis from its upstream steps to fully mature transcripts, we conclude that during the social cycle, tRNA pools are characterized by the expression of nearly all tRNA genes and by changes in abundance of mature tRNAs that reflect the shifts in the expression dynamics of tRNA transcriptional and post-transcriptional processing proteins. Most notably, these changes include a marked overall silencing occurring during the transition from vegetative to streaming stage, rather than in the establishment of the fruiting body.

## Electronic supplementary material

Below is the link to the electronic supplementary material.

Supplementary Material 1

## Data Availability

This study re-analyzes publicly available data. All SRA accession numbers are provided in the Supplementary Methods Table S1.
