# Supplementary material for: Regulation of tRNA expression during the social cycle of the amoeba Dictyostelium discoideum
Source: BMC Mol Cell Biol. 2026 Mar 7;27:19. doi: 10.1186/s12860-026-00580-0 (PMC13063481; doi:10.1186/s12860-026-00580-0)
Supplement: Supplementary file 1 — Supplementary Material 1 [file 12860_2026_580_MOESM1_ESM.pdf]

Supplementary Methods

Supplementary Methods Table S1. List of publicly available libraries used in this study.

Experiment	SRA accession	No. replicate	Stage
ATAC-seq	SRR12137022	1	Vegetative
ATAC-seq	SRR12137023	2	Vegetative
ATAC-seq	SRR12137024	3	Vegetative
ATAC-seq	SRR12137025	1	Streaming
ATAC-seq	SRR12137026	2	Streaming
ATAC-seq	SRR12137027	3	Streaming
ATAC-seq	SRR12137028	1	Mound
ATAC-seq	SRR12137029	2	Mound
ATAC-seq	SRR12137030	3	Mound
ATAC-seq	SRR12137031	4	Mound
ATAC-seq	SRR12137032	1	Fruiting
ATAC-seq	SRR12137033	2	Fruiting
ATAC-seq	SRR12137034	3	Fruiting
RNA-seq	SRR10133967	1	Fruiting
RNA-seq	SRR10133968	2	Fruiting
RNA-seq	SRR10133965	1	Mound
RNA-seq	SRR10133966	2	Mound
RNA-seq	SRR10133963	1	Streaming
RNA-seq	SRR10133964	2	Streaming
RNA-seq	SRR10133961	1	Vegetative
RNA-seq	SRR10133962	2	Vegetative
LOTTE-Seq	SRR11776732	1	Vegetative
LOTTE-Seq	SRR11776731	2	Vegetative
LOTTE-Seq	SRR11776730	1	Streaming
LOTTE-Seq	SRR11776729	2	Streaming
LOTTE-Seq	SRR11776734	1	Fruiting
LOTTE-Seq	SRR11776733	2	Fruiting

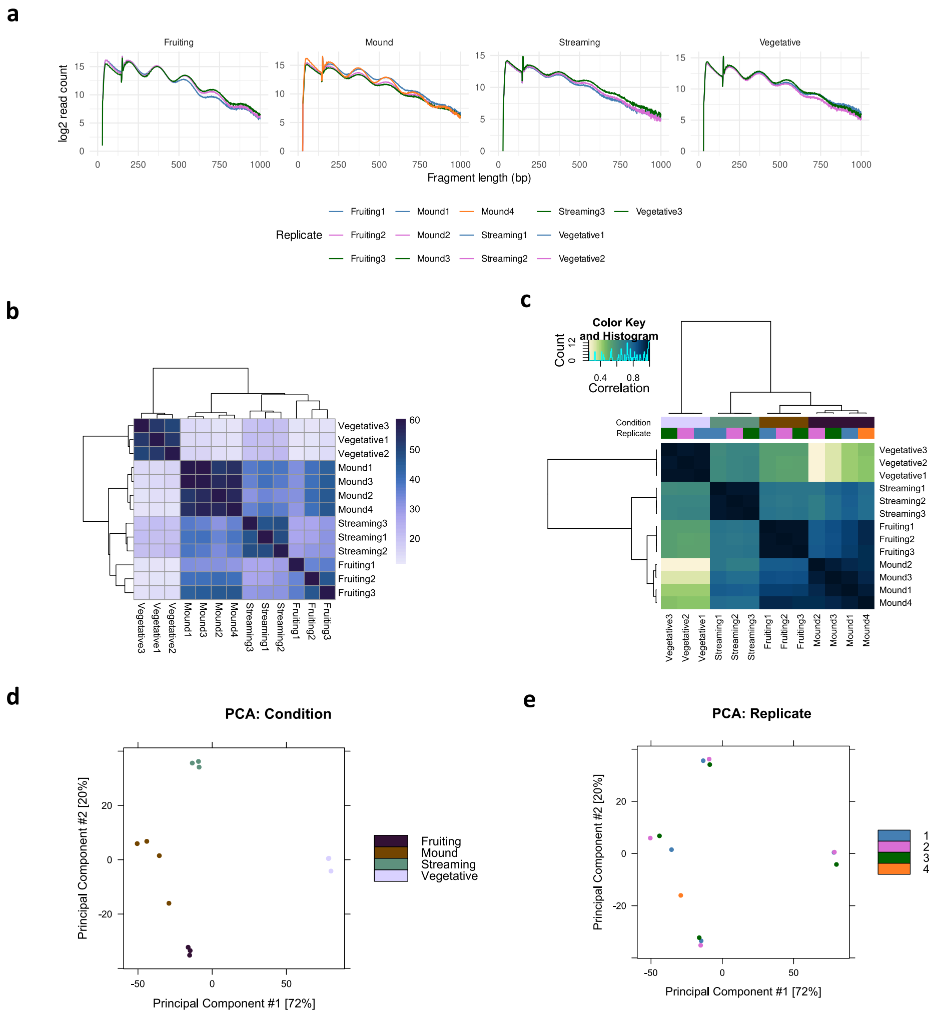

Supplementary Methods Figure 1. Quality check of ATAC-seq libraries. (a) Fragment length distribution clearly showing the nucleosome free regions (peaks bellow 100 bp) and the nucleosome positions in all libraries. (b) Pairwise percentage of IDR replicated peaks. (c) Replicate correlation based on normalized read counts per high confidence peaks. (d) PCA of normalized read count in peaks per replicates. (d) Same PCA as in panel c, but showing the replicate number. Only the two most similar Mound replicates (namely, Mound2 and Mound3) were used in the downstream analyzes.

Supplementary Figures

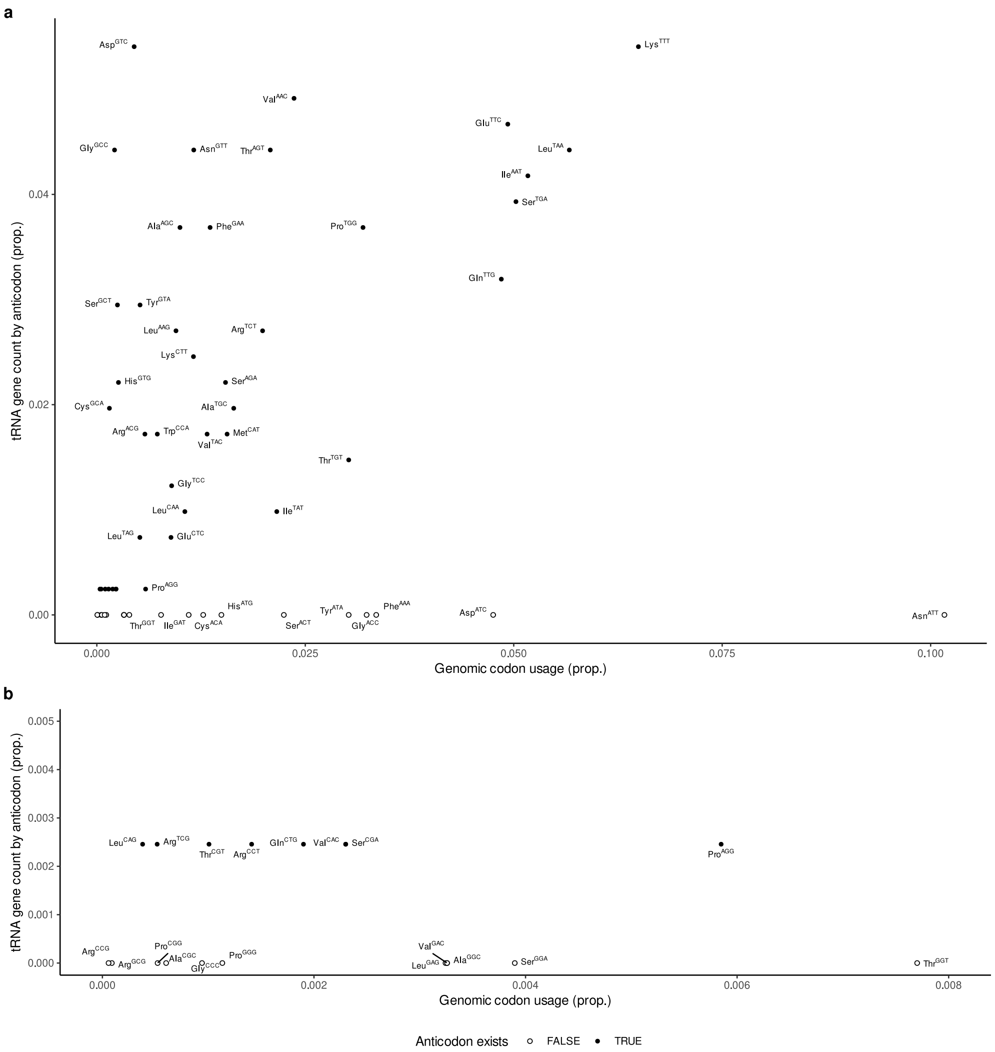

Supplementary Figure 1. Pearson correlation between codon frequency and tRNA gene copy number of isoacceptors. a. Enlarged version of Figure 2b (see main text for description). **b.** Zoom into the cluster of points near the origin (0,0).

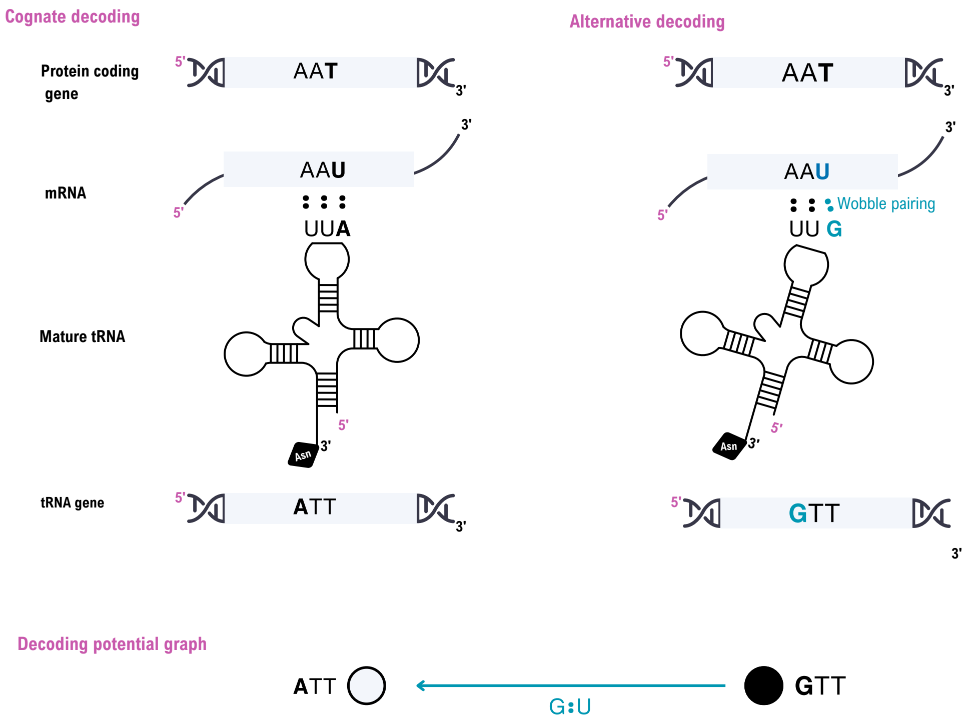

Supplementary Figure 2. Diagram depicting the mechanism represented in the decoding potential graph. Following the example of the main text, we present the two ways in which the codon `AAT` can be decoded. The cognate decoding (top-left panel): in the mRNA this codon will have the sequence `AAU` and will be decoded by the tRNA with anticodon read from 3' to 5', `UUA`. This tRNA was coded in a gene with sequence `ATT`. However, if a tRNA with an `ATT` anticodon does not exist, the codon `AAT` can be alternatively decoded (top-right panel) by a tRNA with anticodon `UUG` (3' to 5'), through a wobble `G:U` anticodon:codon pairing. This alternative tRNA was coded in a gene with anticodon sequence `GTT`. Therefore, in the decoding potential graph, there will be an arrow going from `GTT` to `ATT` with the color of the wobble pairing needed in order to do achieve this alternative decoding (`G:U`).

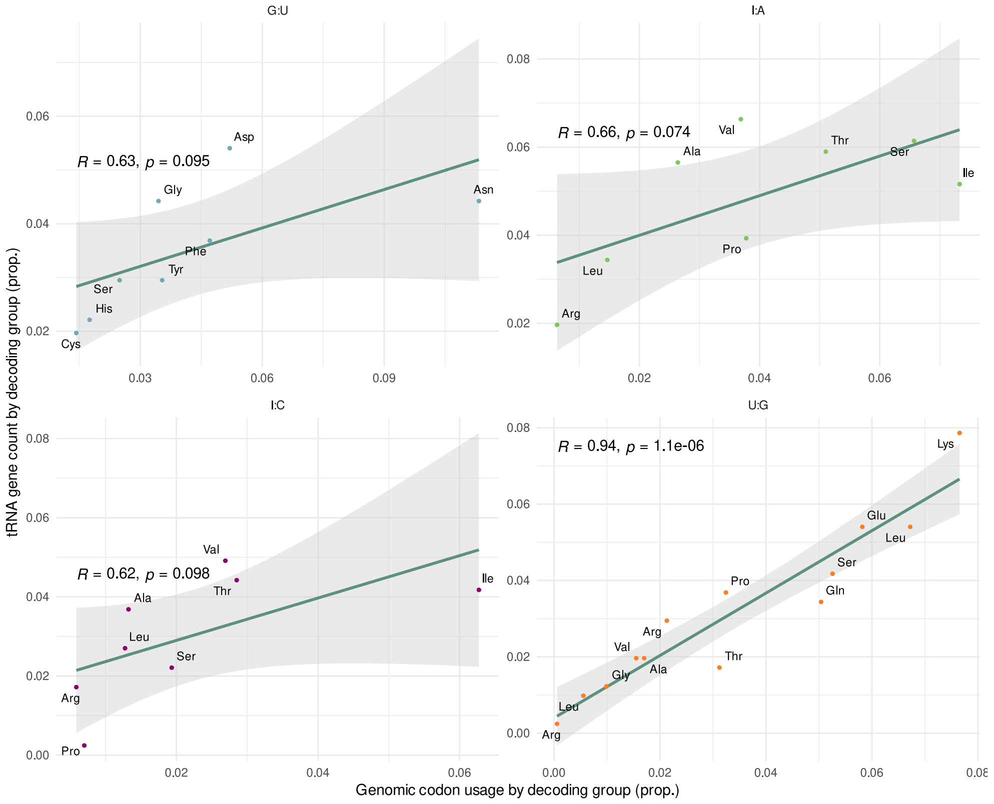

Supplementary Figure 3. Pearson correlation for each wobble pairing group. Pearson correlations between tRNA gene copy numbers and the frequencies of their cognate codons for anticodons linked by the edges of the decoding potential graph. Each panel shows these grouped counts for each type of wobble pairing (G:U), (U:G), (I:A) or (I:C) or, equivalently, for each edge color.

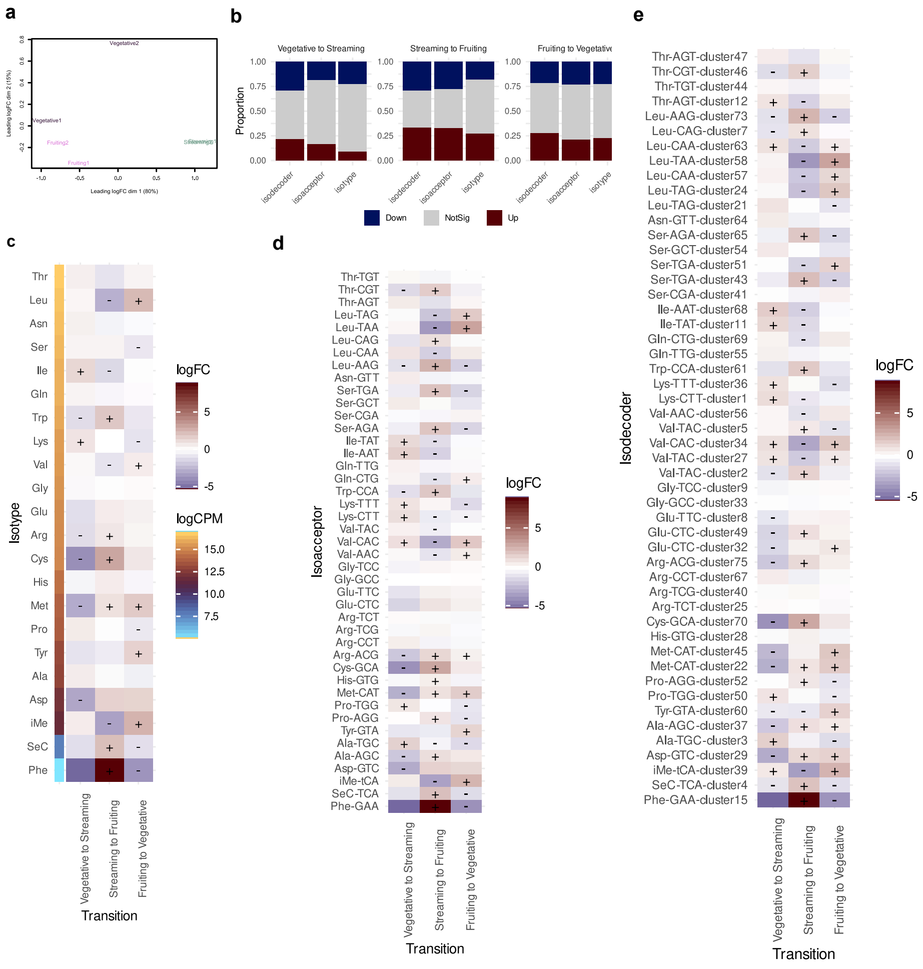

Supplementary Figure 4. Complementary analysis of mature tRNAs expression during the social cycle of *D. discoideum* New experiments on vegetative samples could reveal stronger signals of tRNA expression reactivation from spores to the vegetative state. **a.** Principal component analysis (PCA) of the LOTTE-seq libraries shows that one library is highly similar to the fruiting body samples, which could weaken the signal. The following figures show the results of repeating the analysis after discarding this sample. **b.** Proportion of mature tRNAs exhibiting differential expression levels, shown according to their tRNA class and cycle transition (corresponding raw counts are shown in Sup. Table). Expression patterns of mature tRNA isotypes **c.**, isoacceptors **d.** and isodecoders **e**. The symbols "+" and "-" indicate that a tRNA is statistically significantly up- or down-regulated, respectively. The fold change of tRNA expression logarithmic between two stages (logFC) is indicated by the fill color. As a reference of the abundance of each tRNA isotype in the tRNA pool we show in **c** the read count expressed in logaritmic counts per million scale(logCPM) during the vegetative state.

Supplementary Tables

Chromosome	Chr. length (Mb)	Number of tRNA genes	tRNA gene density (genes/Mb)	Total tRNA gene span (Kb)	Number of coding genes	Coding gene span (Mb)	Coding gene density (genes/Mb)	Ratio tRNA genes to coding genes
Chr6	3.6	86	24.0	6.5	1498	2.5	415.8	0.057
Chr1	4.9	79	16.0	5.8	2019	3.4	410.1	0.039
Chr3	6.4	79	12.0	5.9	2604	4.4	409.6	0.030
Chr2	8.5	90	11.0	6.7	3431	5.9	404.4	0.026
Chr5	5.1	33	6.4	2.4	2030	3.7	396.1	0.016
Chr4	5.5	33	6.1	2.6	2183	3.8	400.5	0.015

Supplementary Table S1. The tRNA gene density density in *D. discoideum*. Table showing the tRNA gene and coding-gene density per chromosome.

Transition	Diff. expression sign	Isotype	Isodecoder	Isoacceptor
Vegetative to Streaming	Not significative	14	31	31
Vegetative to Streaming	Up	3	6	3
Vegetative to Streaming	Down	5	14	9
Streaming to Fruiting	Not significative	15	29	27
Streaming to Fruiting	Up	2	11	8
Streaming to Fruiting	Down	5	11	8
Fruiting to Vegetative	Not significative	21	51	42
Fruiting to Vegetative	Down	1	0	0
Fruiting to Vegetative	Up	0	0	1

Supplementary Table S2. Counts of differentially expressed tRNAs. Number of tRNAs detected in the differential expression test as down-regulated, up-regulated, or not significantly changed, for each tRNA class (isodecoder, isoacceptor or isotype) and for each stage transition.

Transition	Diff. expression sign	Isodecoder	Isoacceptor	Isotype
Vegetative to Streaming	Down	15	8	5
Vegetative to Streaming	Not significative	25	28	15
Vegetative to Streaming	Up	11	7	2
Streaming to Fruiting	Down	15	12	4
Streaming to Fruiting	Not significative	19	17	12
Streaming to Fruiting	Up	17	14	6
Fruiting to Vegetative	Down	11	10	5
Fruiting to Vegetative	Not significative	26	24	12
Fruiting to Vegetative	Up	14	9	5

Supplementary Table S3. Counts of differentially expressed tRNAs in complementary analysis. Number of tRNAs detected in the complementary differential expression test (Sup. Fig. 4) as down-regulated, up-regulated, or not significantly changed, for each tRNA class (isodecoder, isoacceptor or isotype) and for each stage transition.
